# Supplementary figures and images for: The Impact of Constrictotermes cyphergaster (Termitidae: Nasutitermitinae) Termites on Semiarid Ecosystems in Brazil: A Review of Current Research
Source: Insects. 2022 Aug 5;13(8):704. doi: 10.3390/insects13080704 (PMC9409220; doi:10.3390/insects13080704)

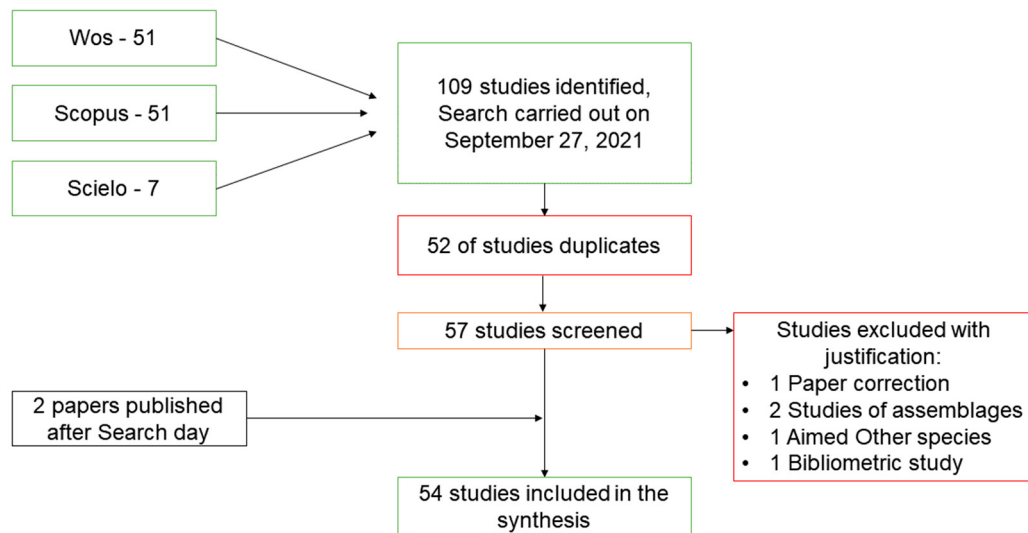

Figure S1: Prism protocol that shows the process to search and choose studies for the synthesis.

Supplement: Supplementary file 1 [file insects-13-00704-s001.zip › insects-1789810-supplementary.pdf]
